# Enhancing microstructural fidelity in near-field high-energy diffraction microscopy reconstruction with Noise2Void denoising

**DOI:** 10.1107/S1600576726007612

**Published:** 2026-09-15

**Authors:** Xiaoxu Guo, Dishant Beniwal, Derek McLain, Jonathan Almer, Jun-Sang Park, Peter Kenesei, Hemant Sharma

**Affiliations:** a Argonne National Laboratory (ANL) 9700 South Cass Avenue Lemont IL 60439 USA; ESRF, France

**Keywords:** near-field high-energy diffraction microscopy, deep learning, image processing, denoising, microstructure, Noise2Void

## Abstract

The resolution and fidelity of grain reconstruction from near-field high-energy diffraction microscopy (NF-HEDM) are often limited by poor signal-to-noise ratios, especially for fine microstructural features. Traditional denoising methods require clean ground-truth data, which is physically impossible in these experiments. To overcome this, we introduce a self-supervised deep learning network, Noise2Void (N2V), to denoise NF-HEDM images at the detector level prior to segmentation. By avoiding the requirement for clean ground-truth images or paired noisy data sets, which are typically unavailable in experimental settings, the N2V technique presented here is highly suitable for NF-HEDM and potentially transferable to other diffraction-based microstructure imaging techniques compromised by uncorrelated noise, such as micro Laue diffraction and far-field HEDM. The N2V model is trained solely with noisy images and then applied to those same images to obtain denoised data. The resulting cleaner images allow for the application of a lower segmentation threshold, enabling the capture of very weak signals that were previously lost. This yields a quantifiable improvement in reconstruction confidence by ∼9% on average and, validated grain by grain against an electron backscatter diffraction (EBSD) ground truth, recovers more of the true grain population – concentrated in the small grains and fine microstructural features that conventional thresholding discards – without altering the boundaries of grains already detected.

## Abstract 2

A self-supervised deep learning model, Noise2Void (N2V), denoises near-field high-energy diffraction microscopy diffraction images without requiring unobtainable clean data. These images allow for lower segmentation thresholds and detection of smaller and weaker diffraction peaks, which are often discarded or overlooked with conventional image processing methods. The result is a ∼9% increase in reconstruction confidence and a more accurate representation of fine microstructural features.

## 1. Introduction

Near-field high-energy diffraction microscopy (NF-HEDM) is a non-destructive method to reconstruct the microstructure of polycrystalline materials with micrometre resolution (Suter *et al.*, 2006 ▸; Li & Suter, 2013 ▸; Lauridsen *et al.*, 2001 ▸; Poulsen, 2003 ▸; Schmidt *et al.*, 2008 ▸; Li *et al.*, 2012 ▸; Liu & Suter, 2020 ▸). Such techniques are well established at large X-ray facilities around the world. Combined with far-field high-energy diffraction microscopy (FF-HEDM), this technique can map the orientations and elastic strain states of grains, and their evolution, in three dimensions inside polycrystalline materials such as metals, ceramics and alloys.

The typical resolution of NF-HEDM is ∼1 µm in position and ∼0.1° in orientation (Suter *et al.*, 2006 ▸; Sparks *et al.*, 2024 ▸). Reconstruction resolution and fidelity are dictated by the ability to detect the shape of diffraction spots with high accuracy, especially for defining the position of grain boundaries. This ability deteriorates as the signal-to-noise ratio deteriorates. For a HEDM experiment, the intensity of the diffraction signal depends on the acquisition time, Bragg angle, structure factor of the diffracting (*hkl*) plane, azimuthal angle and volume of the diffracting grain (Sharma *et al.*, 2012 ▸). Because polycrystalline materials comprise grains with a range of sizes and crystallographic orientations, the intensity of diffraction spots can vary by orders of magnitude within a grain and across different grains. This, combined with the fixed dynamic range and detection efficiency of a given detector, poses a challenge for accurate signal segmentation. From an experimental point of view, the signal-to-noise ratio can be improved by increasing the exposure time or photon flux, which typically results in a higher signal; however, in many cases, exposure times are limited by unavoidable experimental constraints (*e.g.* finite experiment time and the dynamic range of the detector). Thus, efforts to extract meaningful diffraction spot shape and intensity information from noisy diffraction images are crucial to maximize the application of NF-HEDM for understanding material behaviour.

A critical step in NF-HEDM data analysis is the segmentation of diffraction spots from background noise in diffraction images. The traditional approach often involves background subtraction followed by filtering and thresholding. Although effective for sharp spots, this method often struggles with weaker or diffuse spots, characteristic of smaller grain volumes, higher defect content or low structure factors, leading to incomplete or inaccurate grain reconstructions, particularly for features such as small grains or complex twin structures (Suter *et al.*, 2006 ▸; Sharma, 2020 ▸; Sparks *et al.*, 2024 ▸).

In recent years, machine learning methods have shown strong results in the area of X-ray image processing. Examples include locating Bragg peaks faster in FF-HEDM (Liu *et al.*, 2022 ▸) and denoising micro-tomography data using Noise2Inverse (Hendriksen *et al.*, 2020 ▸; Yunker *et al.*, 2025 ▸) or 3D U-Net architectures (Cicek *et al.*, 2016 ▸; Fang *et al.*, 2021 ▸; Hovad *et al.*, 2020 ▸). However, the success of most of these models is predicated on the availability of clean ground-truth images or multiple noisy instances of the same underlying signal, which are often impractical or impossible to obtain in typical NF-HEDM experiments.

In this paper, we employ the Noise2Void (N2V) model (Krull *et al.*, 2018 ▸), a self-supervised deep learning technique, to overcome the limitations in NF-HEDM data analysis. By comparing our results with the well characterized ‘ground-truth’ data set from Sparks *et al.* (2024 ▸), which was meticulously generated using multi-modal serial sectioning with high-precision electron backscatter diffraction (EBSD), we demonstrate that the N2V approach yields enhanced reconstruction results by removing background noise while preserving weak diffraction signals. This leads to marked improvements in the final microstructure reconstruction, including a significant increase in overall confidence, the formation of physically realistic grain and twin boundaries, and a more accurate capture of the small-grain population. We show that this method successfully overcomes key limitations of traditional analysis and provides a robust pathway to enhance microstructural fidelity from NF-HEDM.

## 2. Data and methods

### 2.1. NF-HEDM data acquisition and pre-processing

Experimental details for the acquisition of NF-HEDM diffraction images, used in this work, are available in the report by Sparks *et al.* (2024 ▸). The NF-HEDM technique has been described in detail elsewhere (Suter *et al.*, 2006 ▸; Li *et al.*, 2012 ▸). Here, we summarize the key experimental details and initial processing steps common to both methods.

The experiments were completed on the 1-ID beamline at the Advanced Photon Source (APS) at Argonne. Monochromatic X-rays were produced by a standard APS undulator and monochromated using a cryogenically cooled bent double Laue monochromator (Shastri *et al.*, 2002 ▸; Shastri, 2004 ▸) optimized specifically for high-energy X-rays. For the data set used here, the 71.68 keV incident beam was focused with refractive sawtooth lenses (Shastri, 2004 ▸) to a line 1.5 µm tall by 2 mm wide. The focused beam impinged on a sample fabricated from low solvus high refractory (LSHR) nickel-based superalloy. The sample cross section illuminated by the focused beam was 1 × 1 mm, nominally. For NF-HEDM, the detector converts the diffracted X-rays to visible light by a scintillator placed directly behind the sample, and the visible light was collected on a camera system with 2048 × 2048 pixels, each 1.48 µm square. The diffraction images were collected at two sample-to-detector distances of ∼5 and 9 mm from the rotation axis and the sample. Diffraction images were collected during continuous rotation of the sample about the vertical axis (ω) from −90° to 90°, integrating over 0.25° intervals, resulting in 720 images per detector distance.

**Figure 1:**
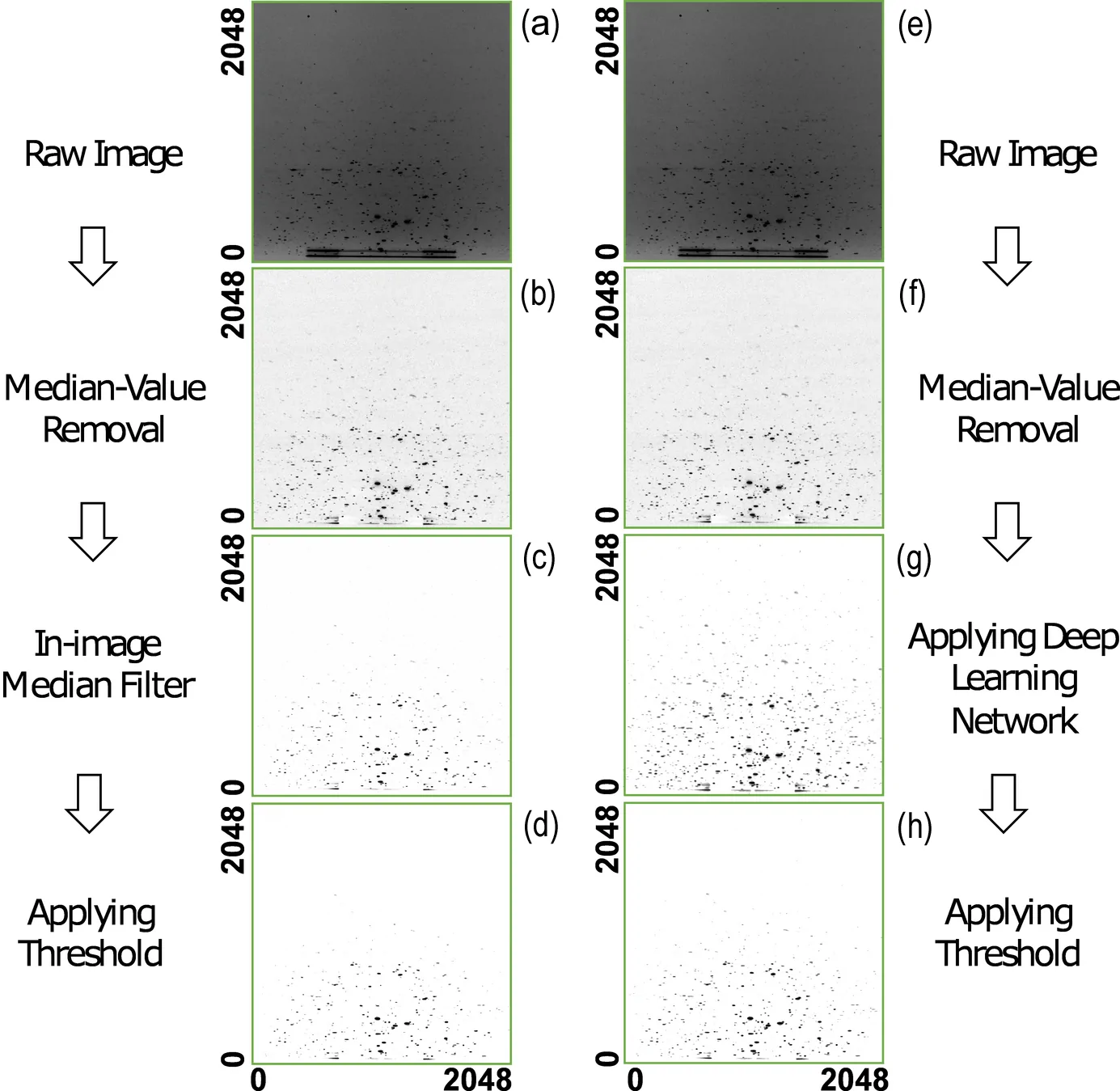
Comparison of image processing workflows. (*a*) Raw NF-HEDM detector image (ω = 6.75°). (*b*) Image after median removal (common pre-processing step, input to traditional path). (*c*) Traditional path after background subtraction (ten counts) and in-image median filter. (*d*) Traditional path, final binary image after thresholding. (*e*) Same raw image as panel (*a*). (*f*) Image after median removal (input to N2V path). (*g*) N2V path, image after applying trained N2V model. (*h*) N2V path, final binary image after thresholding (three counts).

Figs. 1 ▸(*a*) and 1(*e*) show an example raw NF-HEDM detector image collected from the sample; intensity is shown in greyscale, where darker denotes higher intensity and lighter denotes lower intensity. The raw detector images contain regions corresponding to diffracted beams superimposed on background noise. Part of the background is generated by stray scattering (especially from components like the beam stop) and potentially stray visible light. These background features are relatively stable during sample rotation in ω; hence, median-value removal is commonly employed as a common pre-processing step. We generate and subtract a median image, calculated pixel-wise over the entire 180° rotation range. Pixels with negative values after subtraction are set to zero. Figs. 1 ▸(*b*) and 1(*f*) show the image after this median removal. While the diffraction spots are now more prominent and some large-scale background (like the beam-stop shadow) is reduced, significant low-level background noise persists. This median-removed image serves as the starting point for both the traditional and the N2V processing pipelines.

### 2.2. Traditional diffraction spot segmentation

Traditional data analysis aims to convert processed diffraction images into binarized signals, identifying pixels belonging to diffraction spots (True) versus background (False), which are then used for reconstruction (Suter *et al.*, 2006 ▸; Sharma, 2020 ▸; Sharma *et al.*, 2026*a* ▸). Following median removal [Fig. 1 ▸(*b*)], the standard routine involves two steps:

(i) Filtering. An in-image median filter (*e.g.* 3 × 3 kernel) is applied to reduce residual noise. Further smoothing using filters assuming Poisson and/or Gaussian noise might also be applied.

(ii) Thresholding. Pixels with intensities above a fixed threshold (ten counts in this study) are considered part of a diffraction spot.

The parameters used here (3 × 3 median filter, threshold of ten counts) were selected based on experience as providing a reasonable balance between noise removal and signal preservation for this type of NF-HEDM data, serving as a representative traditional baseline. Fig. 1 ▸(*c*) shows the image after background subtraction and median filtering, and Fig. 1 ▸(*d*) shows the final binary image obtained after thresholding. A key limitation here is the empirical choice of the threshold and filter parameters, which often need careful tuning for different data sets and may still discard weak signals or retain noise.

### 2.3. Noise2Void denoising and segmentation

The new approach uses the N2V deep learning method (Krull *et al.*, 2018 ▸) for image denoising after the initial median removal [Fig. 1 ▸(*f*)]. Deep learning methods for image denoising can be broadly classified as follows:

(i) Supervised methods (*e.g.* convolutional neural networks). Require training pairs of noisy input images (*x*^*i*^) and their corresponding clean target images (*s*^*i*^). These methods are generally not applicable here as clean ground-truth NF-HEDM images are physically unobtainable.

(ii) Self-supervised methods. Do not require clean targets.

(*a*) *Noise2Noise* (Lehtinen *et al.*, 2018 ▸). Requires pairs of independently degraded versions of the same underlying signal (*s* + *n*, *s* + *n*′). Acquiring such pairs with constant signal (*s*) and independent noise (*n*, *n*′) during an NF-HEDM scan is impractical.

(*b*) *Blind-spot methods* [*e.g.* Noise2Self (N2S) (Batson & Royer, 2019 ▸), N2V (Krull *et al.*, 2018 ▸)]. Can train on single noisy images under the assumption that noise is pixel-wise independent (or nearly so). These methods train a network to predict a pixel’s true value based on its neighbourhood, excluding the central pixel itself (the ‘blind spot’).

In the case of N2S, we observed that it sometimes led to artefacts in the shapes of the denoised diffraction spots, probably due to the spatial correlations inherent in diffraction spots which conflict with the pixel-wise independence assumption of N2S. N2V provided more consistent and reliable denoising results.

The N2V model for NF-HEDM workflow is implemented in the open-source *MIDAS-NF-preProc* Python package, which builds on the *CAREamics* implementation of N2V (Krull *et al.*, 2019 ▸). We used 1440 median-subtracted images (720 from each detector distance) for training and inference. The network architecture used is a U-Net (Cicek *et al.*, 2016 ▸) of depth 2, kernel size 3, with batch normalization, using 32 initial feature maps, and a linear activation function in the last layer. For efficient training, we followed the N2V method­ology: input patches of 64 × 64 pixels were used, with *N* = 64 pixels randomly selected per patch for the blind-spot manipulation during training. The effective receptive field size was 5 × 5 (Krull *et al.*, 2018 ▸). We used a batch size of 128, an initial learning rate of 0.0004 and the standard CSBDeep learning rate schedule (Weigert *et al.*, 2018 ▸). The model was trained for a maximum of 100 epochs, stopping earlier if the validation loss ceased to decrease. Training took approximately 5 min per detector data set (720 images) using an NVIDIA RTX A6000 GPU. Applying the trained model to denoise each 2048 × 2048 image took less than 1 s.

Fig. 1 ▸(*g*) shows the denoised image after applying the trained N2V model to the input image in Fig. 1 ▸(*f*). Crucially, because N2V effectively removes background noise [compare Figs. 1 ▸(*g*) and 1 ▸(*f*)], a much lower intensity threshold can be applied for segmenting diffraction peaks. For this work, we applied a simple intensity threshold of three counts to the N2V-denoised image to generate the final binary image [Fig. 1 ▸(*h*)] in contrast to ten counts for the traditional workflow. Attempting to apply a threshold of three to the traditional workflow results in an overwhelming number of false-positive pixels due to background noise, rendering the subsequent reconstruction computationally intractable and highly inaccurate. Therefore, N2V denoising is the crucial enabling step that allows this lower threshold to be used, retaining weaker diffraction signals that would inevitably be eliminated by the higher effective threshold required in the traditional method.

### 2.4. Grain reconstruction

Based on the binary image output from either the standard routine [Fig. 1 ▸(*d*)] or the N2V pipeline [Fig. 1 ▸(*h*)], grain structures were reconstructed using the *MIDAS* software package (Sharma, 2020 ▸; Sharma *et al.*, 2026*a* ▸; Sharma *et al.*, 2026*b* ▸). The parameters for the grain reconstruction algorithm itself were fixed for both reconstructions. Thus, any differences in the reconstructed microstructure depend solely on the method used for generating the binary diffraction images. Notably, while the N2V method increases the number of valid ‘True’ pixels by recovering weak signals, it simultaneously eliminates a vast number of spurious noise pixels. As a result, the computational time required for the forward-modelling reconstruction remains comparable to that of the traditional method but it yields a significantly higher fidelity output.

Reconstruction was performed using the forward modelling technique described by Suter *et al.* (2006 ▸), Sharma (2020 ▸) and Sharma *et al.* (2026*a* ▸). The physical sample volume was tessellated with an equilateral triangular grid of 3 µm side length. These triangles were assigned crystallographic orientations based on an optimization procedure that maximizes the overlap between simulated diffraction images (based on the triangle’s position and orientation) and the experimental binary diffraction images. The procedure optimized each triangle independently. The reconstruction confidence for each triangle is defined as the ratio of the number of its simulated peaks that match observed peaks in the experimental binary data to the total number of simulated peaks expected for that orientation assignment. Triangles are then coloured by orientation (*e.g.* using Euler angles mapped to RGB space) or by their confidence value.

## 3. Results and discussion

### 3.1. Background removal and spot segmentation: traditional versus N2V

**Figure 2:**
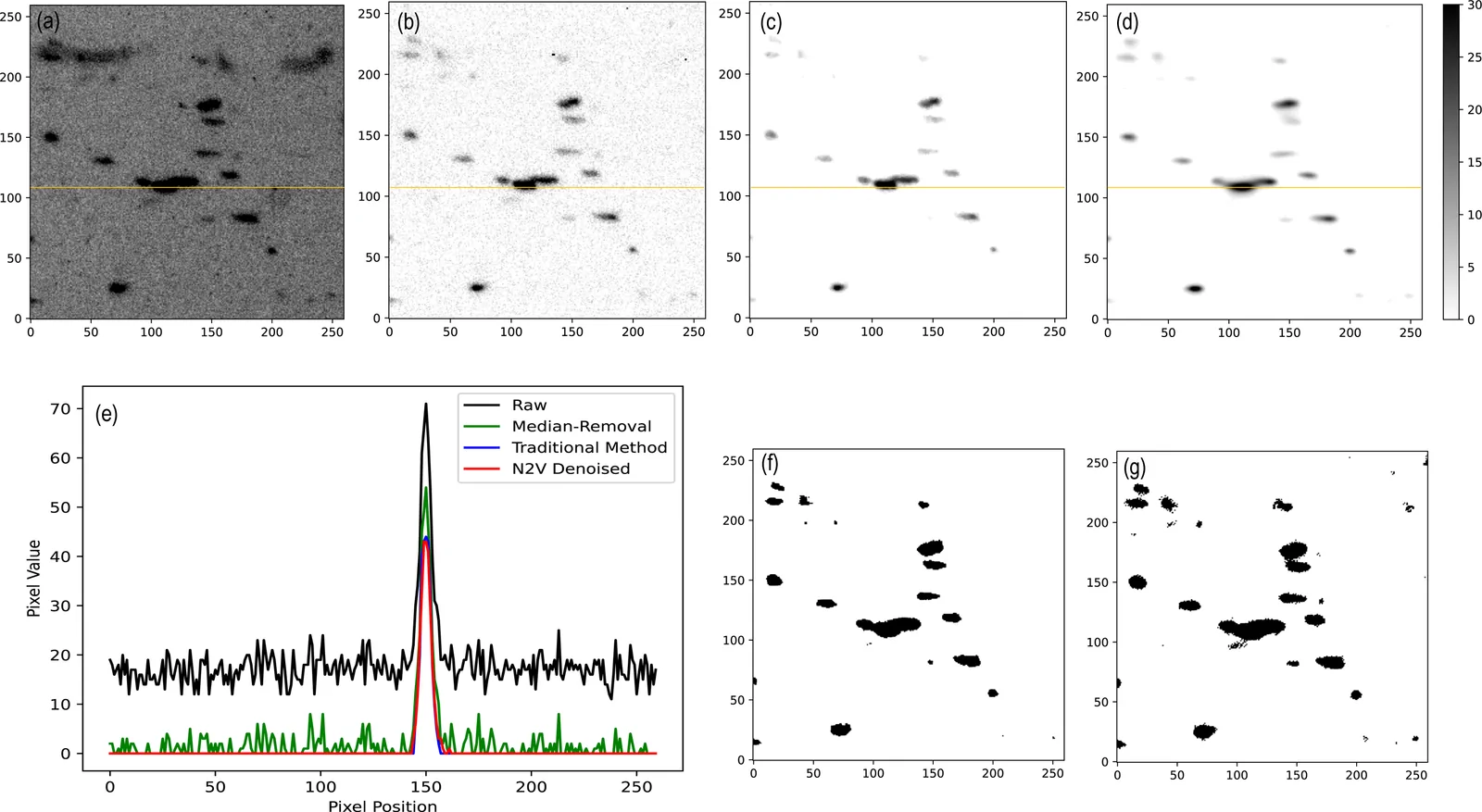
An enlarged comparison of image processing steps. (*a*) Raw image snippet. (*b*) After median removal. (*c*) Traditional result (after subtraction and median filter). (*d*) N2V denoised result. (*e*) Pixel intensity profiles along the yellow line indicated in panels (*a*)–(*d*) for raw (black), median-removed (green), traditional (blue) and N2V denoised (red). (*f*) Binary segmentation result from the traditional method. (*g*) Binary segmentation result from the N2V method. Note the recovery of the weak lower spot in panel (*g*) compared with panel (*f*).

Figs. 1 ▸(*c*)–1 ▸(*d*) and 1 ▸(*g*)–1 ▸(*h*) illustrate the intermediate and final steps of the traditional and N2V image processing workflows, respectively, starting from the median-removed image [Figs. 1 ▸(*b*) and 1 ▸(*f*)]. Fig. 2 ▸ provides enlarged views for detailed comparison.

In detail, Fig. 2 ▸(*a*) shows the raw image snippet. and Fig. 2 ▸(*b*) shows the same snippet after median removal. Note the persistence of low-level noise and some ‘cloudy‘ artefacts.

Fig. 2 ▸(*c*) shows the result after applying the traditional routine (3 × 3 in-image median filter, threshold of ten counts). Noise is reduced, but spot intensities are also affected.

Fig. 2 ▸(*d*) shows the result after applying the N2V denoising. The background appears much cleaner and smoother compared with both Figs. 2 ▸(*b*) and 2 ▸(*c*).

Fig. 2 ▸(*e*) presents quantitative pixel-value profiles along the horizontal yellow line indicated in Figs. 2 ▸(*a*)–2 ▸(*d*). The raw data (black) show a noisy background. Median removal (green) reduces large-scale variations but leaves noise. The traditional method (blue) further smooths the background [the blue curve is virtually indistinguishable from the green (median-removed) curve, except at the edges of the peak]. The N2V denoised profile (red) shows the smoothest background while retaining peak definition.

Figs. 2 ▸(*f*) and 2 ▸(*g*) show the final binary segmentation results from the traditional and N2V methods, respectively. The difference is striking. In the traditional result [Fig. 2 ▸(*f*)], the smaller spot below the main central spot is almost entirely lost. In contrast, the N2V result [Fig. 2 ▸(*g*)], obtained using a lower threshold (three counts) on the cleaner data [Fig. 2 ▸(*d*)], clearly recovers this weak spot with a size comparable to its appearance in the raw data [Fig. 2 ▸(*a*)]. Furthermore, Fig. 2 ▸(*g*) reveals more complete signal at the edges of stronger spots than Fig. 2 ▸(*f*).

This visual comparison demonstrates a key limitation of the traditional method: its reliance on empirical thresholding and filtering often leads to the loss of weak signals. Upon close examination of many images, it is found that, using the traditional procedure, weaker spots or weaker areas connected to stronger spots are frequently missing or incomplete. The N2V method, by effectively learning and removing the complex noise structure first, allows for a more sensitive threshold to be applied and conserves the very small peaks crucial for the accurate reconstruction of fine microstructural features, such as small grains or twin lamellae.

### 3.2. Comparison of the reconstructed grain structures

In this section, we compare the microstructure reconstruction results using the two diffraction image processing and segmentation workflows. The comparison begins at the image processing level, demonstrating the fundamental difference in signal preservation. We then show how this initial difference propagates through the final reconstructed microstructure, leading to quantifiable improvements in reconstruction confidence, grain size statistics and the physical accuracy of challenging microstructural features.

#### 3.2.1. Confidence, grain orientation maps and grain statistics

**Figure 3:**
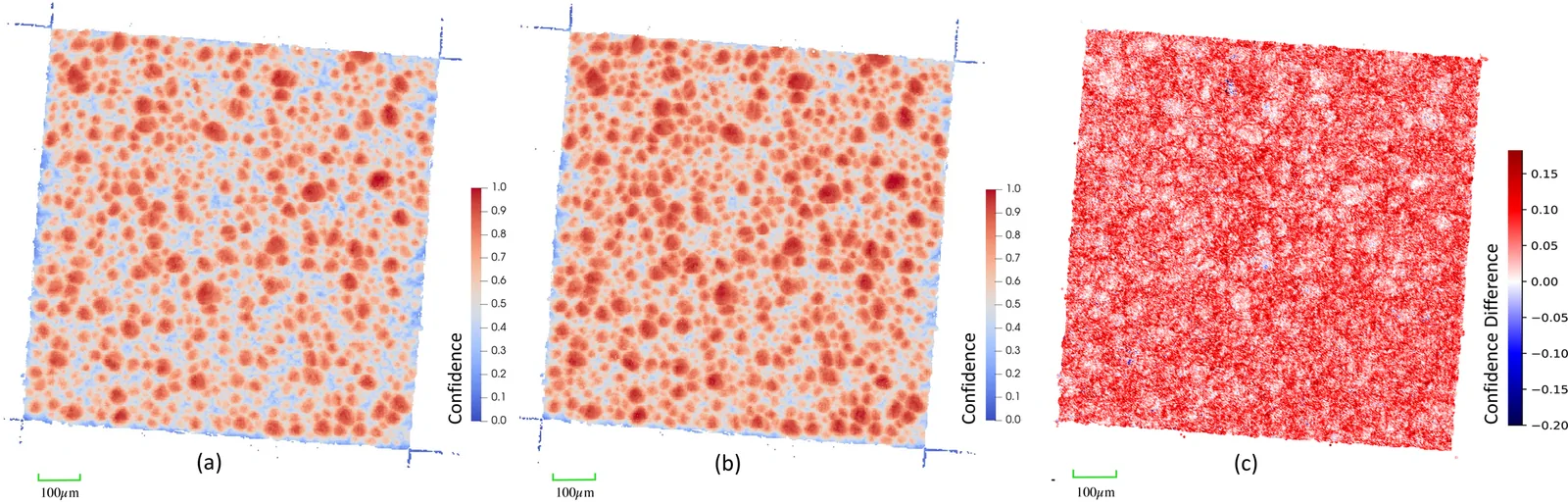
The N2V processing method yields a quantifiable increase in reconstruction confidence. (*a*) Confidence map from the traditional method and (*b*) from the N2V method. The N2V result shows visibly higher confidence values (more yellow and orange tones). (*c*) The pixel-wise difference map (N2V − Traditional) illustrates the improvement. The predominantly red colouring indicates a widespread increase in confidence when using the N2V method, with the average gain measured at 9%.

Fig. 3 ▸ compares the confidence maps using diffraction data processed with (*a*) the traditional method (threshold value ten, in-image median filter) and (*b*) the N2V method (threshold value three and no in-image median filter). Higher confidence values (closer to one) indicate a better match between the assigned orientation and the observed diffraction data. The overall grain structure appears similar, indicated by the high confidence locations in Figs. 3 ▸(*a*) and 3 ▸(*b*). However, when the two maps are subtracted (N2V − Traditional), a widespread increase in confidence is readily visible when the N2V method is employed in the image processing [Fig. 3 ▸(*c*)]. The average increase in confidence across the entire map is ∼9%.

It is important to address the concern that a lower segmentation threshold inherently generates more foreground pixels, which could in principle inflate overlap-based confidence metrics simply by increasing the probability of a random spatial match (*i.e.* by dilating spots rather than recovering true signal). We test this directly in Section 3.4 and find the opposite: at a matched threshold N2V does not enlarge spots; it removes the background that otherwise makes a low threshold unusable, and the additional spots it recovers carry the spatial and rocking-curve signatures of genuine high-*Q* Bragg reflections. Together with the grain-level validation against the EBSD ground truth (Section 3.3), this confirms that the 9% increase reflects a genuine recovery of diffraction signal rather than a thresholding artefact.

**Figure 4:**
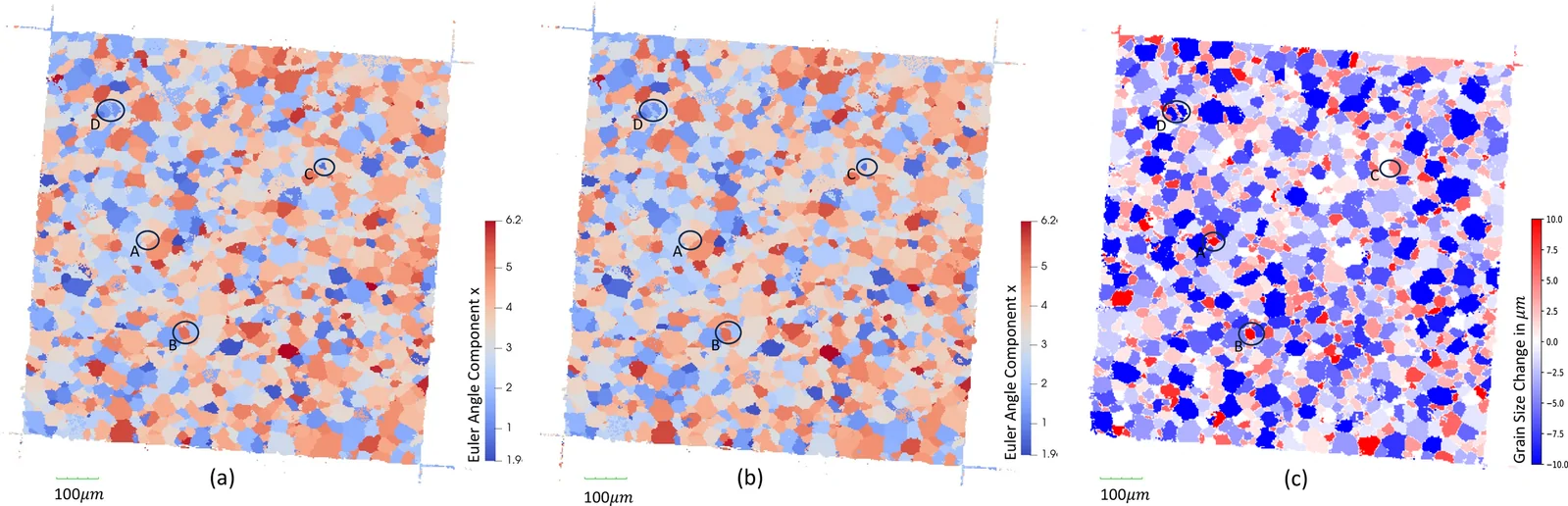
N2V processing corrects grain shapes and reveals a systematic resizing trend. (*a*) An orientation map from the traditional method is compared with (*b*) the N2V-based map, which shows important changes in grain shape and indexing. (*c*) The grain size difference map (N2V size − Traditional size) illustrates the key trend. Red indicates that smaller grains are systematically reconstructed as larger with the N2V method, while blue indicates that some larger grains are slightly reduced in size at their boundaries. This is not an artefact but a correction, as the N2V method better captures the weak diffraction signals from smaller volumes. A representative grain is examined in Fig. 6 ▸.

Fig. 4 ▸ shows the grain orientation maps reconstructed using (*a*) the traditional method and (*b*) the N2V method, coloured by a component of the Euler angle. Although the maps are largely consistent, differences in grain indexing (assigning orientation) and the resulting grain shapes are visible upon close examination. Throughout this work the reconstruction is a single two-dimensional layer (a ∼1.5 µm thick section defined by the line-focused beam); ‘grain size’ therefore refers to the grain’s *cross-sectional area* (or its area-equivalent diameter) in that section, not a three-dimensional volume. A grain is defined operationally as a contiguous region of voxels whose orientations lie within 1° of one another (with spatial proximity additionally required for spatially separate grains; see below). Fig. 4 ▸(*c*) displays the change in cross-sectional area between the N2V and traditional reconstructions for each identified grain. White indicates no change with N2V, red an increase and blue a decrease. A clear trend emerges: smaller grains tend to appear larger (red) in the N2V reconstruction, while some larger grains appear slightly smaller (blue).

These trends – smaller grains reconstructed slightly larger and some boundaries becoming smoother – illustrate that the improved segmentation from N2V leads not only to higher confidence but also to more physically plausible grain shapes, particularly by better resolving smaller features and interfaces; they are quantified systematically against the EBSD ground truth in Section 3.3.

Table 1 ▸ shows the number of grains found in the two reconstructions. An orientation is unique if the smallest misorientation angle between the orientation of a set of voxels and that of any other voxel in the reconstruction is greater than 1°. Spatially separate grains were computed by including the physical proximity of the voxels and using the same 1° criterion for misorientation. The N2V-based workflow consistently identifies a higher number of both spatially separate grains (3109) and unique orientations (1918 ▸) than the traditional reconstruction. To make a like-for-like comparison with the ground truth, Table 1 ▸ reports the EBSD grain count for the *same reconstructed layer* [4496 grains, from the equal-volume layer in Sparks *et al.* (2024 ▸)]. Previous HEDM analysis of this data set using traditional segmentation recovered only ∼1600 grains; with algorithmic and parameter optimizations the traditional count here rose to 2880, and to 3109 with N2V. The residual difference relative to EBSD is not a seeding limitation: the FF-HEDM grain list used to seed the reconstruction contains 3775 orientations covering ∼95% of the EBSD grains, and ∼90% of the grains the near-field reconstruction misses were in fact seeded (Section 3.3). The deficit is instead a near-field *detection* limit concentrated in the smallest grains; as quantified below, recovery exceeds 88% for grains above 200 µm^2^ in cross section and approaches 100% for the largest grains. N2V improves recovery specifically in this small/intermediate grain regime.

#### 3.2.2. Grain size distribution

**Figure 5:**
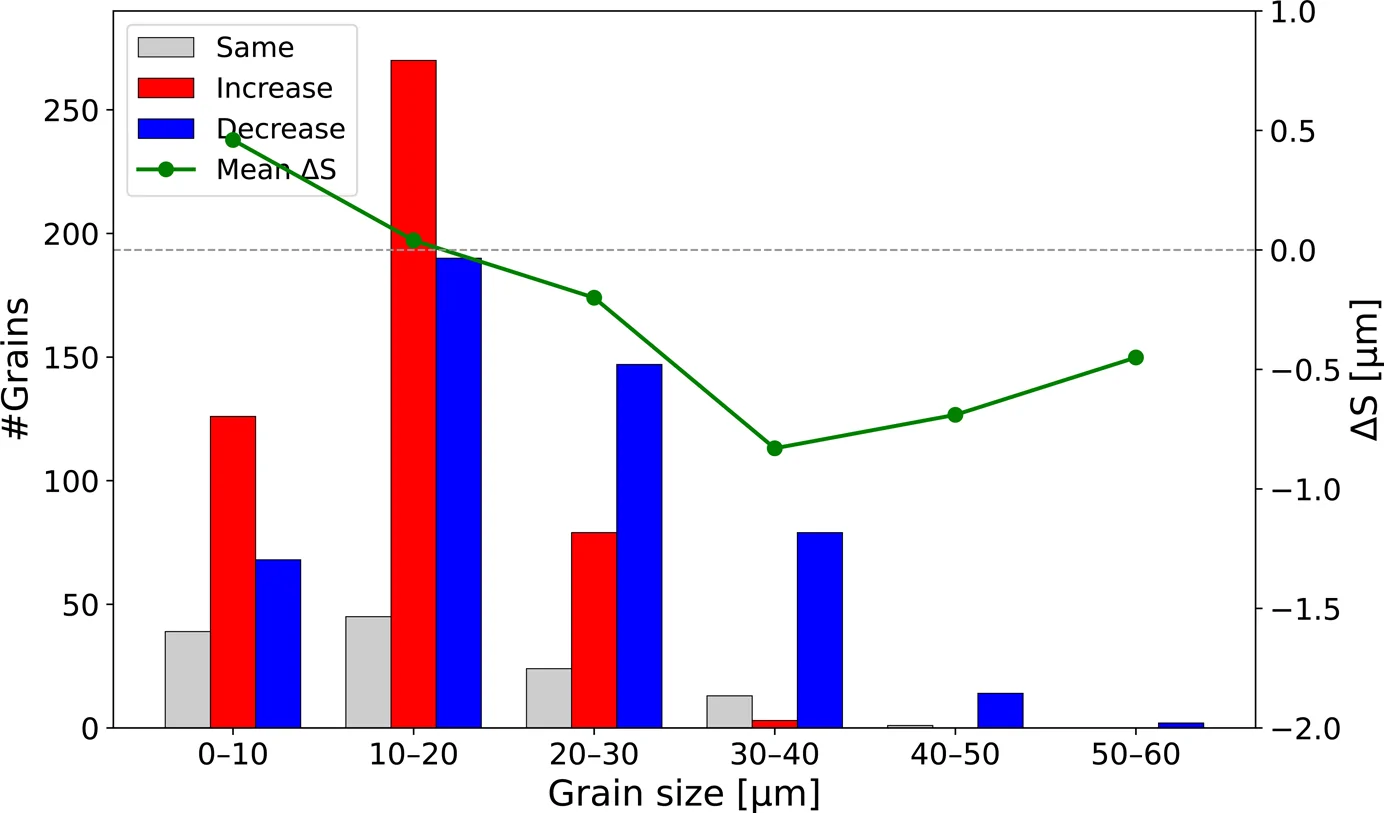
Quantitative analysis confirms that the N2V method preferentially recovers the size of smaller grains. The histogram plots the change in grain size for the N2V reconstruction relative to the traditional method (reference). The data clearly show that for grains smaller than 20 µm, a larger number of grains are reconstructed as larger (red bars) than smaller (blue bars). The green line (right axis), representing the mean size change, confirms this trend. This indicates a correction of the systematic undersizing of small grains that occurs in traditional processing, and a slight erosion of large grain boundaries.

Fig. 5 ▸ quantifies the changes in grain size distribution. The histogram bins grains based on their size in the traditional reconstruction (reference). Within each bin, it shows the number of grains that remained the same size (grey), increased in size (red) or decreased in size (blue) in the N2V reconstruction. For grains smaller than 20 µm (especially 10–20 µm), a larger number of grains increased in size (red) than decreased (blue). For grains larger than 20 µm, the trend reverses, with more grains decreasing in size (blue).

The green line in Fig. 5 ▸ shows the mean change in size within each bin, measured against the right-hand *y* axis. It is positive for smaller grains (peaking in the 0–10 µm bin) and becomes negative for grains larger than 30 µm.

This quantitatively confirms the observed trend: the N2V method, by better retaining small/weak diffraction spots, leads to an apparent increase in the size of smaller grains and a slight reduction at the boundaries of larger grains during reconstruction. This phenomenon highlights the zero-sum nature of the tessellated reconstruction grid; as weaker signals from small grains are successfully preserved and mapped, they outcompete and reclaim boundary triangles that were previously misassigned to adjacent larger grains due to low signal. This ultimately results in a more accurate overall representation of the microstructure.

To understand the underlying mechanism for this improvement, we must move beyond global statistics and analyse the local performance of the reconstruction at its most challenging points: the interfaces between grains.

#### 3.2.3. From improved segmentation to physically accurate grain boundaries

To understand precisely how the N2V method leads to improved mapping, especially at challenging interfaces, we must examine the confidence values for adjacent grains. The comprehensive ‘ground-truth’ data set from Sparks *et al.* (2024 ▸) is essential for validation. This data set provides a reliable benchmark as it was constructed using serial sectioning with high-precision EBSD, an electron microscopy technique that offers superior spatial resolution and direct measurement of surface crystallography. It therefore serves as an ideal reference against which HEDM reconstruction fidelity can be judged, and it also highlights the inherent difficulties of traditional HEDM reconstruction in accurately resolving fine microstructural features like coherent twin boundaries.

**Figure 6:**
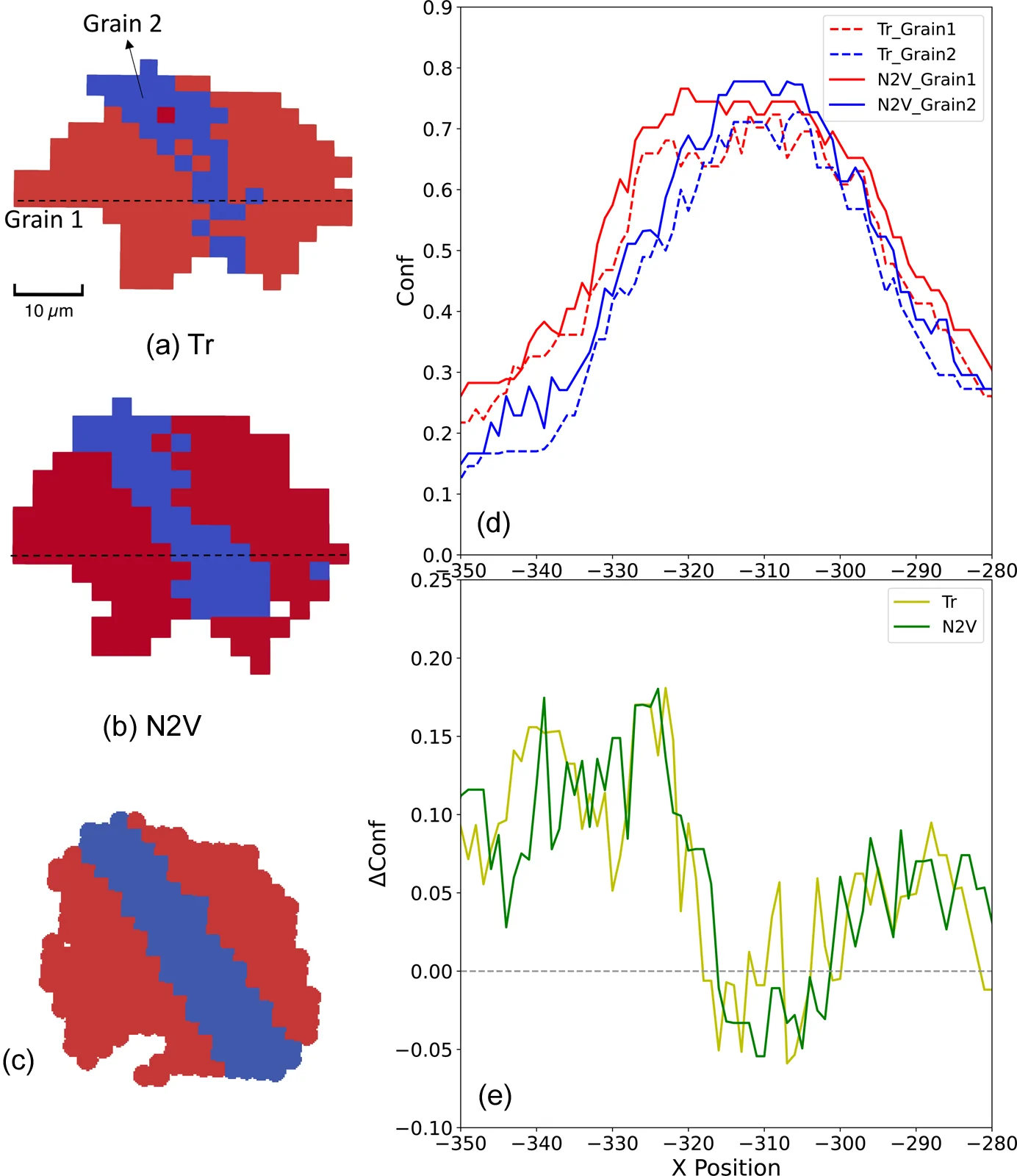
The N2V method produces physically realistic twin boundaries. (*a*) The traditional reconstruction results in a non-physical jagged boundary with erroneous ‘islands’. (*b*) The N2V reconstruction correctly identifies a flat crystallographically consistent twin boundary. (*c*) The corresponding ground-truth EBSD map confirms the true morphology of the twin boundary, matching the N2V result closely. (*d*) Line-scan analysis along the dashed line shows the N2V method yields consistently higher confidence values. (*e*) The confidence difference plot highlights the key improvement: the N2V method (green) produces a single sharp zero crossing, indicating a well defined boundary, while the traditional method (yellow) produces a noisy transition indicative of spatial uncertainty.

Fig. 6 ▸ provides a direct comparison for such a boundary. The traditional reconstruction method [Fig. 6 ▸(*a*)] produces a jagged interface containing erroneous ‘islands’ of the parent grain within the twin, a non-physical representation. This artefact arises from the aggressive empirically tuned filtering that discards weak diffraction signals near the interface as noise. In stark contrast, the N2V-processed data yield a twin boundary that is remarkably flat and continuous [Fig. 6 ▸(*b*)], which is far more consistent with the expected crystallography of annealing twins and closely matches the EBSD ground truth [Fig. 6 ▸(*c*)].

This visual improvement is quantified in the line-scan analysis. As shown in Fig. 6 ▸(*d*), the confidence values obtained using N2V (solid lines) are consistently higher than those from the traditional method (dashed lines) across the entire interface. More importantly, the confidence difference plot [Fig. 6 ▸(*e*)] shows the N2V method (green line) produces a single clean zero crossing, indicating a well defined boundary location. The traditional method’s noisy transition (yellow line) corresponds directly to the spatial uncertainty of the jagged boundary.

This single twin is an illustrative example; the systematic grain-by-grain validation of these improvements against the EBSD ground truth – including the finding that, for grains recovered by both pipelines, boundary positions are statistically unchanged – is presented next (Section 3.3). Because N2V is self-supervised, *i.e.* trained directly on the noisy images themselves, requiring no clean target, it is applicable by construction to any NF-HEDM data set; we demonstrate it here on one well characterized sample with EBSD ground truth and, in Section 3.5, verify that the method transfers to a second material of different (hexagonal) crystal symmetry without retuning.

### 3.3. Quantitative validation against the EBSD ground truth

The preceding comparisons are qualitative or anecdotal. Because the same sample was characterized by serial-sectioned EBSD (Sparks *et al.*, 2024 ▸), we can validate the reconstructions quantitatively, grain by grain, against the ground truth. The N2V and traditional reconstructions of the layer were registered to the corresponding EBSD section by a point-set registration of grain centroids, refined jointly on centroid position and crystal misorientation (cubic symmetry); the resulting spatial transform is a near-identity similarity. We additionally segmented the registered EBSD layer into grains (3° tolerance, minimum-sized cut), yielding ∼3900 grains, consistent with the 4496 grains of Sparks *et al.* (2024 ▸) at their segmentation parameters. A reconstructed grain is counted as recovered when an EBSD grain lies within a size-adaptive radius and agrees in orientation to better than 5°.

#### 3.3.1. Grain recovery versus size

**Figure 7:**
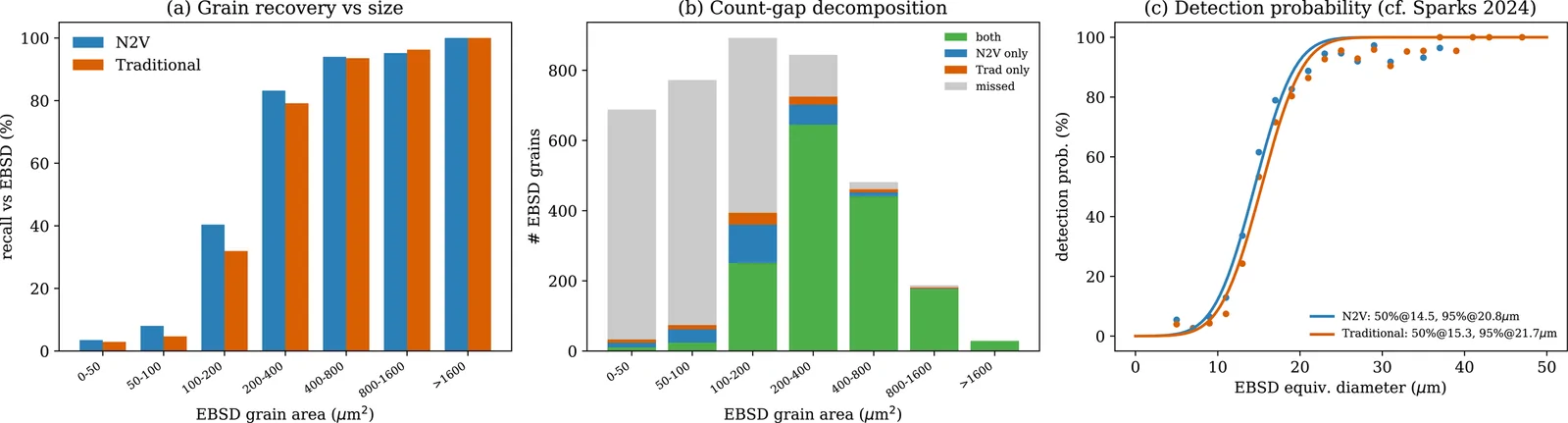
Quantitative grain recovery against the EBSD ground truth. (*a*) Recall as a function of EBSD grain cross-sectional area for the N2V and traditional pipelines: both recover essentially all large grains, while N2V’s advantage is concentrated in the small-to-intermediate range (50–400 µm^2^). (*b*) Decomposition of the EBSD grain population by outcome and size (recovered by both, by N2V only or by traditional only, or missed by both); the grains missed by both are overwhelmingly the smallest – the quantitative answer to why NF-HEDM recovers only a fraction of the EBSD grains. (*c*) Grain-detection probability versus size, following the analysis framework of Sparks *et al.* (2024 ▸) (their Fig. 13): the fitted error function slopes reproduce the reported behaviour to ∼1%, and N2V shifts the curve toward smaller grains. Absolute diameters are 2D cross-sectional and so sit below the 3D volume-equivalent values of Sparks *et al.* (2024 ▸).

N2V recovers 1807 EBSD grains and the traditional pipeline 1668 [Fig. 7 ▸(*a*)]. Crucially, both pipelines confirm against EBSD at the *same* rate (∼94% of reconstructed grains have an EBSD counterpart), so N2V’s additional grains are real rather than spurious. The recovery is strongly size-dependent [Fig. 7 ▸(*a*)]: both methods recover >94% of grains larger than 400 µm^2^ and approach 100% for the largest grains, while recovery falls steeply below ∼200 µm^2^. N2V’s advantage is concentrated in the small-to-intermediate range (*e.g.* 40% versus 32% recovery at 100–200 µm^2^), exactly where weak Bragg signals are discarded by a high threshold. This is the quantitative form of the long-standing observation that NF-HEDM ‘sees only a fraction’ of the EBSD grains: the missing population is overwhelmingly the smallest grains, near the line beam and voxel resolution limit, and not a different boundary population [Fig. 7 ▸(*b*)].

#### 3.3.2. The deficit is a detection limit, not a seeding limit

Because the near-field reconstruction is seeded from an FF-HEDM orientation list, that list bounds what can be recovered. The far-field list contains 3775 orientations and covers ∼95% of the EBSD grains; of the grains missed by *both* near-field segmentations, ∼90% were nonetheless present as candidate seeds. The count gap is therefore inherited from the near-field detection of weak small-grain signals – precisely the regime N2V targets – rather than from incomplete seeding. Casting the recovery probability as a function of grain size [Fig. 7 ▸(*c*)] reproduces the error function detection curve reported for this data set by Sparks *et al.* (2024 ▸) (the fitted curve slopes agree to within ∼1%) and shows that N2V shifts the curve toward smaller grains, lowering the 50% and 95% detection sizes.

#### 3.3.3. Orientation accuracy

**Figure 8:**
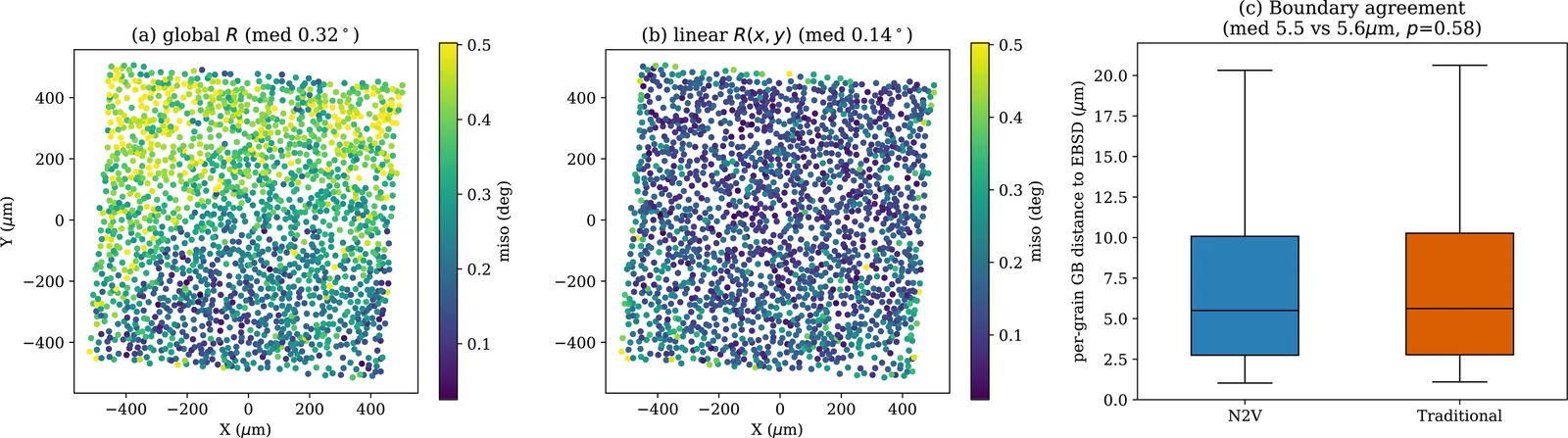
Orientation accuracy and boundary agreement for recovered grains. (*a*) Misorientation to EBSD with a single global reference rotation, showing a systematic in-plane gradient of ∼0.6° mm^−1^ across the EBSD scan. (*b*) After a position-dependent reference rotation the residual is flat at a median of 0.14°. This gradient independently reproduces the systematic EBSD orientation error documented for this data set by Sparks *et al.* (2024 ▸). (*c*) Per-grain boundary distance to EBSD for grains recovered by *both* pipelines: the N2V and traditional distributions are statistically indistinguishable (combined medians 5.5 versus 5.6 µm; Mann–Whitney *p* = 0.58), showing that N2V does not perturb the boundaries of commonly detected grains.

For recovered grains, the reconstructed orientation agrees with EBSD to a median of 0.14° [Figs. 8 ▸(*a*) and 8 ▸(*b*)] after accounting for a systematic in-plane orientation gradient of ∼0.6° mm^−1^ across the EBSD scan. This gradient is a known EBSD effect [it independently reproduces the systematic EBSD orientation error of ∼1.7° plus pattern-centre drift documented for this data set by Sparks *et al.* (2024 ▸)]; after its removal the residual is flat across grain size, at the level of EBSD’s own absolute accuracy. The accuracy of recovered grains is essentially identical for the two pipelines.

#### 3.3.4. N2V adds grains without perturbing the existing boundary network

A natural concern is that the changes wrought by N2V might imply an entirely different population of boundaries. They do not: for grains recovered by *both* pipelines, the per-grain boundary distance to EBSD is statistically identical [median 5.5 µm for N2V versus 5.6 µm for traditional; Mann–Whitney *p* = 0.58; Fig. 8 ▸(*c*)]. N2V leaves the boundaries of commonly detected grains in place and adds small grains; correspondingly, the per-grain cross-sectional areas of commonly detected grains agree equally well with EBSD for both methods. The improvement N2V delivers is in *completeness* – recovering weak small-grain and twin signals – rather than in the geometric precision of grains that traditional analysis already finds.

### 3.4. N2V recovers true diffraction signal without over-dilating spots

**Figure 9:**
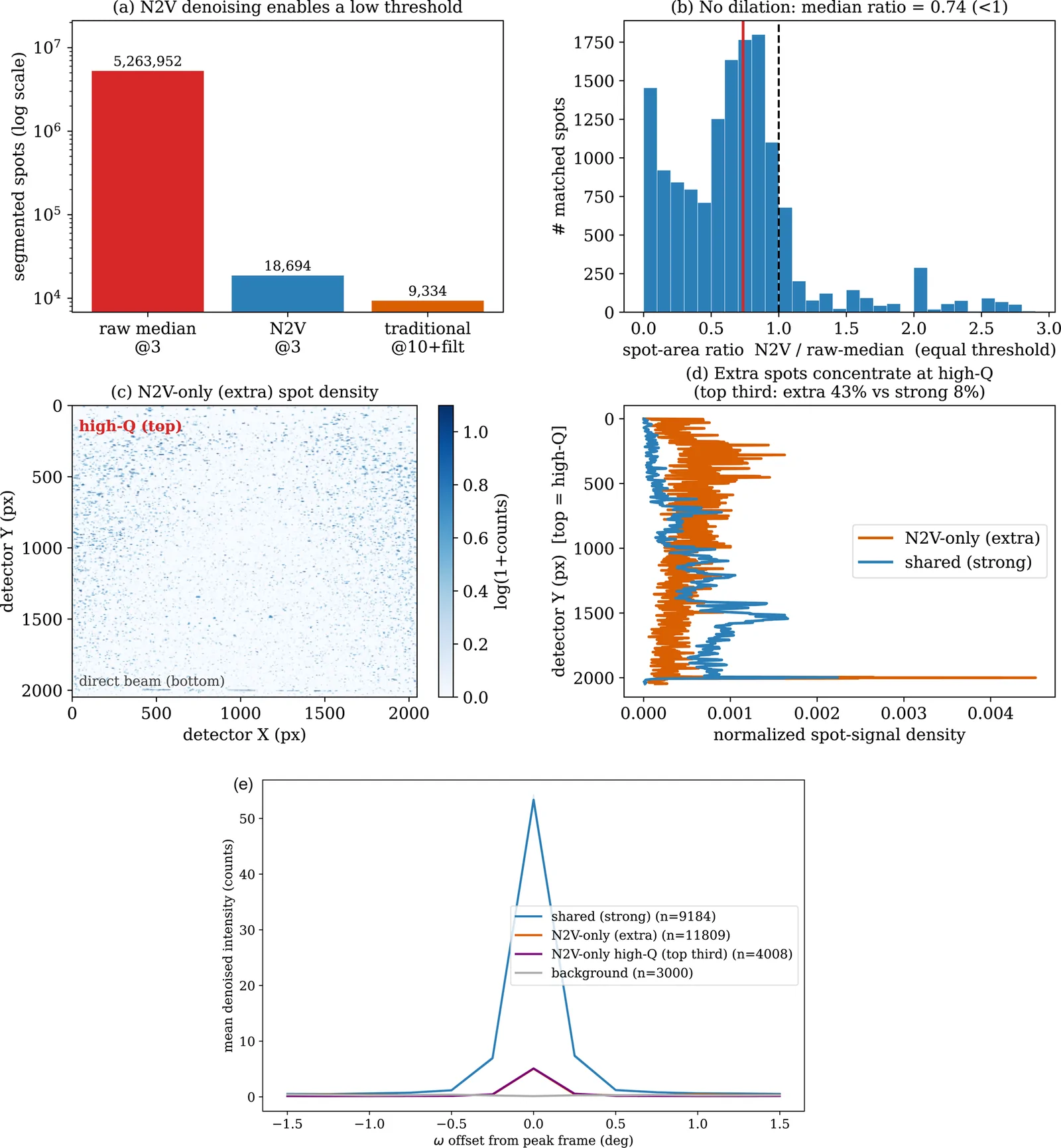
N2V recovers true diffraction signal without over-dilating spots. (*a*) Segmented spot counts: thresholding the raw median-removed image at three counts yields ∼10^6^ (noise-dominated) spots, versus ∼10^4^ for the N2V-denoised image and the traditional pipeline – N2V removes the noise that makes a low threshold unusable. (*b*) At a matched threshold, N2V spot areas are slightly smaller than raw (median ratio 0.74): denoising does not enlarge spots. (*c*)–(*d*) The additional spots recovered only by N2V are concentrated at the high-*Q* (top) region of the detector, where the atomic form factor suppresses higher-order reflections. (*e*) Mean intensity profile across ω (rocking curve), peak-aligned: the N2V-recovered high-*Q* spots show a coherent rise-then-fall profile like strong spots, the signature of genuine Bragg reflections rather than single-frame noise.

The confidence gain (Fig. 3 ▸) could in principle arise from N2V dilating diffraction spots, which would inflate overlap-based metrics. Three independent spot-level tests on the real data show this is not the case. First, at a *matched* threshold the N2V-denoised spots are if anything slightly smaller than those segmented from the raw median-removed image (median area ratio 0.74), so the denoising does not enlarge spots. The reason N2V enables a much lower threshold is noise removal, not dilation: thresholding the raw median-removed image at three counts yields of the order of 10^6^ spurious peaks, whereas the N2V-denoised image yields ∼10^4^ (Fig. 9 ▸). Second, the additional spots recovered only by N2V are not spatially random, as noise would be; they concentrate at the high-*Q* (top) region of the detector (Fig. 9 ▸), exactly where the atomic form factor suppresses higher-order reflections below the traditional detection limit – the same region where simulated-but-unobserved signal was reported for this data set by Sharma *et al.* (2026*b* ▸). Third, these recovered high-*Q* spots exhibit a coherent rise-then-fall intensity profile across adjacent ω frames [Fig. 9 ▸(*e*)], the rocking-curve signature of a genuine Bragg reflection, rather than appearing in a single frame as noise would. Together these establish that N2V recovers physically real weak high-*Q* diffraction signal that the traditional threshold discards, rather than artificially growing spots.

### 3.5. Generality to a second material and crystal system

To test whether the N2V denoising is a general-purpose pre-processor rather than specific to the LSHR nickel superalloy, we applied the identical self-supervised procedure to a Ti–7Al specimen of hexagonal symmetry (space group No. 194) – a different crystal system, reconstructed with the same *MIDAS* methodology (Sharma, 2020 ▸; Sharma *et al.*, 2026*a* ▸). As an independent co-located EBSD volume is not available for this sample, we used a clean high signal-to-noise ratio reconstruction as the reference (it reproduces the established reconstruction orientations to 0.05°) and injected additive read-noise of increasing magnitude (σ = 15, 30 and 45 counts) into the raw detector images. Each noisy data set was reconstructed twice: once with the standard median/threshold pre-processing and once with N2V denoising trained directly on the noisy frames.

**Figure 10:**
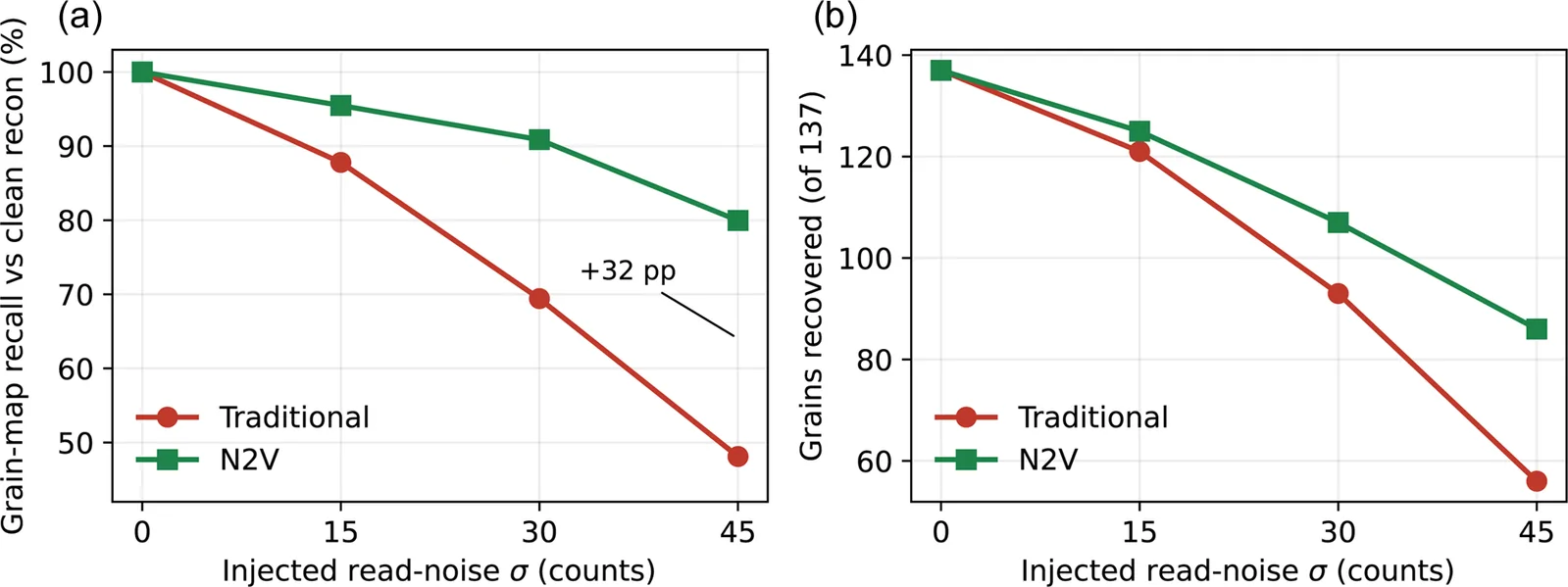
Generality of N2V denoising to a second material and crystal system (Ti–7Al, hexagonal, space group No. 194). A clean high signal-to-noise ratio reconstruction is used as the reference and additive read-noise of increasing magnitude (σ) is injected into the raw detector images. (*a*) Grain-map recall (fraction of reference voxels recovered within 5°) versus injected noise for the traditional and N2V pipelines. (*b*) The number of grains recovered (of 137). The N2V advantage grows with noise, while the orientation accuracy of the recovered voxels is identical between the methods (∼0.05° median) – improved completeness, not precision, reproducing the LSHR finding on a different crystal system.

The recall of the clean grain map degrades far more slowly for the N2V branch (Fig. 10 ▸). At σ = 30 counts the traditional pipeline recovers 69% of the confidently indexed voxels and 93 of 137 grains, whereas N2V recovers 91% of the voxels and 107 grains; at the most severe level (σ = 45) the gap widens to 80% for N2V versus 48% for the traditional pipeline (86 versus 56 grains). Crucially, the orientation accuracy of the recovered voxels is identical between the two methods (median misorientation ∼0.05°): exactly as for LSHR, the N2V benefit is improved *completeness* of the recovered microstructure, not a change in the accuracy of the orientations that are recovered. The advantage grows monotonically with noise and, as expected for a self-supervised method that requires no clean target, transfers to this second material and crystal system without any material-specific retuning. We note that this is a controlled noise-injection test against a clean internal reference, rather than a second independent ground-truth measurement; it isolates the effect of the denoising on reconstruction completeness as the signal-to-noise ratio is varied. We emphasize that the single-sample scope of the ground-truth validation reflects the scarcity of NF-HEDM data sets with independent co-located ground truth (such as the serial-sectioned EBSD used here) that can be shared in an open publication, rather than any limitation of the method. Because N2V is trained on each data set’s own noisy images – requiring no clean target and no per-data set retraining – it is directly applicable to the further NF-HEDM data sets available to us that could not be included in this study, and we anticipate applying it broadly as suitable benchmark data become shareable.

### 3.6. Broader implications for microstructural fidelity

The collective results presented here highlight how the N2V approach fundamentally solves the ‘thresholding dilemma’ inherent in traditional HEDM analysis. Traditional methods force a difficult compromise: a low threshold retains weak signals from small grains but also incorporates significant background noise, while a high threshold produces a clean reconstruction but erases the very features that are often of greatest interest. Our N2V-based workflow decouples these problems by first removing the noise, allowing for a subsequent low threshold to be applied with confidence.

This is not just a theoretical improvement. The comprehensive analysis by Sparks *et al.* (2024 ▸) used co-located EBSD to show that traditional HEDM reconstruction systematically fails to capture a significant fraction of the small-grain population present in the ground truth. The grain-by-grain comparison in Section 3.3 confirms this bias and quantifies the N2V improvement against the ground truth: N2V recovers 1807 EBSD grains versus the traditional method’s 1668 at equal (∼94%) confirmation purity, with the gain concentrated in the small-grain regime where traditional thresholding fails. The N2V-based reconstruction therefore provides a more complete map of the true grain population, while leaving the boundaries of commonly detected grains unchanged.

This has two profound implications for materials science. First, by more accurately capturing the small-grain population (Fig. 5 ▸), it prevents artificial skewing of grain size distributions. In studies of phenomena like grain growth or recrystallization, mischaracterizing the population of small grains can lead to erroneous conclusions about the underlying kinetics. The ability to detect grains reliably down to a few micrometres provides a statistically sound basis for such analysis.

Second, for the weak features that traditional analysis fails to resolve, N2V recovers more physically plausible boundaries (Fig. 6 ▸); we emphasize that for grains both pipelines already detect, boundary positions are unchanged (Section 3.3), so the benefit is the recovery of previously missed features rather than a global sharpening of boundaries. This matters for materials modelling: the morphology of a recovered twin boundary, for instance, dictates how dislocations interact with it, governing slip transmission and strain hardening, and the non-physical jagged interfaces produced when a weak twin is poorly segmented are problematic inputs for high-fidelity simulations such as crystal plasticity finite-element modelling. By recovering these features as smooth crystallographically consistent boundaries, the N2V workflow yields microstructural maps that are more complete and more suitable as simulation inputs. The full potential of this higher-quality input will be realized when it is paired with reconstruction algorithms that make use of the full greyscale intensity information, as discussed below.

### 3.7. Methodological considerations and a vision for future reconstruction

While our N2V workflow significantly improves the input for reconstruction, the process still culminates in a binarization step. This conversion of a rich greyscale image into a binary map represents a fundamental loss of information. All quantitative intensity data, which contain subtle details about spot shape, peak overlap and boundary positions, are discarded in favour of a simple on/off threshold. This is the final empirical bottleneck in an otherwise increasingly sophisticated analysis pipeline.

The ultimate goal for the field, enabled by high-quality denoising like that shown here, is to move beyond binarization entirely. We propose a future direction centred on ‘direct intensity reconstruction’. In such a framework, the reconstruction algorithm would not match binary images but would instead seek to optimize a forward model that directly reproduces the denoised greyscale diffraction images.

This paradigm shift would unlock several key advantages previously considered out of reach:

(i) Sub-voxel boundary localization. By fitting the intensity gradient across an interface, the model could place a grain boundary with a precision greater than the reconstruction voxel size itself, moving from a discrete to a quasi-continuous representation of the microstructure.

(ii) Improved deconvolution of overlapping peaks. Instead of treating overlapping spots as a single binary region, a direct intensity model could fit the sum of multiple simulated diffraction profiles to the complex measured intensity, allowing for more robust separation of grains that are close in orientation and position.

(iii) A truly quantitative confidence metric. The goodness-of-fit could be measured with a true statistical metric (*e.g.* a chi-squared value), replacing the current semi-quantitative ‘overlap fraction’ and providing a far more robust measure of confidence in the assigned orientation.

Achieving this long-term vision requires several intermediate steps. First, building on the two crystal systems demonstrated here, the robustness of N2V denoising should be validated across a still wider range of materials and experimental conditions. Second, releasing a pre-trained N2V model to the HEDM community would be an invaluable service, democratizing access to high-fidelity denoising and accelerating the development of the next-generation reconstruction algorithms that can use this enhanced data quality.

The principles of this denoising workflow are not limited to NF-HEDM. Because N2V learns the underlying signal structure from the noise statistics, it is well suited to a range of characterization techniques where data are often noise-limited. For example, in FF-HEDM and micro Laue diffraction, this method could improve the precision of weak diffraction peak fitting, leading to more accurate orientation and strain measurements. In electron microscopy, N2V could denoise low-dose TEM images of beam-sensitive materials or improve the indexing of noisy EBSD images. Furthermore, it could be applied to denoise the projection data in X-ray computed tomography (XCT) prior to reconstruction, a common strategy for reducing artefacts and improving the clarity of the final 3D volume.

## 4. Conclusions

We have successfully applied a Noise2Void self-supervised deep learning model to denoise NF-HEDM diffraction images, overcoming key limitations of traditional analysis. This data-driven approach is highly practical, as it is trained directly on noisy experimental data without requiring unobtainable clean ground-truth images. The N2V pipeline effectively removes complex background noise, allowing for a lower and more sensitive segmentation threshold.

This leads to measurable improvements in the subsequent grain reconstruction, demonstrated by an average 9% increase in confidence and, validated grain by grain against the EBSD ground truth, the recovery of more of the true grain population (1807 versus 1668 EBSD-confirmed grains at equal confirmation purity), concentrated in the small grains that traditional thresholding discards. The recovered weak features, including twin lamellae, are reconstructed as more physically plausible boundaries, while the boundaries of grains common to both pipelines are left unchanged – so the gain is in completeness rather than in the geometric precision of already-detected grains. These more complete microstructural maps are better-suited inputs for advanced simulations such as crystal plasticity models, where a complete small-grain population and faithful twin geometry support more predictive modelling of material behaviour. This work helps narrow the fidelity gap between X-ray and electron-based microstructure characterization.

## Figures and Tables

**Table 1:** Number of grains found in the reconstruction, compared on an equal-volume basis with the EBSD ground truth Incorporating N2V nearly doubles the number of grains recovered compared with early traditional benchmarks, closing the gap with the EBSD ground truth (Sparks *et al.*, 2024 ▸).

Method	Spatially separate grains	Unique orientations
EBSD ground truth (this work†)	4496	–
N2V (this work)	3109	1918
Traditional (this work)	2880	1763
Traditional (previous work‡)	∼1600	–

† EBSD count for the same reconstructed layer (equal-volume).

‡ Previous HEDM analysis of this same data set prior to algorithmic optimizations (Sparks *et al.*, 2024 ▸).
